# Comparison of evoked potentials across four related developmental encephalopathies

**DOI:** 10.1186/s11689-023-09479-9

**Published:** 2023-03-04

**Authors:** Joni N. Saby, Sarika U. Peters, Timothy A. Benke, Shannon M. Standridge, Lindsay C. Swanson, David N. Lieberman, Heather E. Olson, Alexandra P. Key, Alan K. Percy, Jeffrey L. Neul, Charles A. Nelson, Timothy P. L. Roberts, Eric D. Marsh

**Affiliations:** 1 Division of Radiology Research, Children’s Hospital of Philadelphia, Philadelphia, PA USA; 2 Department of Pediatrics, Vanderbilt University Medical Center, Vanderbilt Kennedy Center, Nashville, TN USA; 3 Department of Pediatrics, Neurology,, Pharmacology and Otolaryngology, University of Colorado School of Medicine and Children’s Hospital Colorado, Aurora, CO USA; 4 Division of Neurology, Cincinnati Children’s Hospital Medical Center, University of Cincinnati College of Medicine, Cincinnati, OH, USA; 5 Department of Neurology, Boston Children’s Hospital, Boston, MA USA; 6 Department of Hearing and Speech Sciences, Vanderbilt University Medical Center, Vanderbilt Kennedy Center, Nashville, TN USA; 7 Department of Pediatrics (Neurology), University of Alabama at Birmingham, Birmingham, AL USA; 8 Laboratories of Cognitive Neuroscience, Boston Children’s Hospital, Boston, MA USA; 9 Department of Pediatrics, Harvard Medical School, Graduate School of Education, Harvard University, Cambridge, MA USA; 10 Division of Child Neurology, Children’s Hospital of Philadelphia, Abramson Research Building- Room 502E, 3615 Civic Center Boulevard, Philadelphia, PA 19104 USA; 11 Orphan Disease Center, Perelman School of Medicine, University of Pennsylvania, Philadelphia, PA USA

## Abstract

**Background:**

Developing biomarkers is a priority for drug development for all conditions, but vital in the rare neurodevelopmental disorders where sensitive outcome measures are lacking. We have previously demonstrated the feasibility and tracking of evoked potentials to disease severity in Rett syndrome and CDKL5 deficiency disorder. The aim of the current study is to characterize evoked potentials in two related developmental encephalopathies, MECP2 duplication syndrome and FOXG1 syndrome, and compare across all four groups to better understand the potential of these measures to serve as biomarkers of clinical severity for the developmental encephalopathies.

**Methods:**

Visual and auditory evoked potentials were acquired from participants with MECP2 duplication syndrome and FOXG1 syndrome across five sites of the Rett Syndrome and Rett-Related Disorders Natural History Study. A group of age-matched individuals (mean = 7.8 years; range = 1–17) with Rett syndrome, CDKL5 deficiency disorder, and typically-developing participants served as a comparison group. The analysis focused on group-level differences as well as associations between the evoked potentials and measures of clinical severity from the Natural History Study.

**Results:**

As reported previously, group-level comparisons revealed attenuated visual evoked potentials (VEPs) in participants with Rett syndrome (*n* = 43) and CDKL5 deficiency disorder (*n* = 16) compared to typically-developing participants. VEP amplitude was also attenuated in participants with MECP2 duplication syndrome (*n* = 15) compared to the typically-developing group. VEP amplitude correlated with clinical severity for Rett syndrome and FOXG1 syndrome (*n* = 5). Auditory evoked potential (AEP) amplitude did not differ between groups, but AEP latency was prolonged in individuals with MECP2 duplication syndrome (*n* = 14) and FOXG1 syndrome (*n* = 6) compared to individuals with Rett syndrome (*n* = 51) and CDKL5 deficiency disorder (*n* = 14). AEP amplitude correlated with severity in Rett syndrome and CDKL5 deficiency disorder. AEP latency correlated with severity in CDKL5 deficiency disorder, MECP2 duplication syndrome, and FOXG1 syndrome.

**Conclusions:**

There are consistent abnormalities in the evoked potentials in four developmental encephalopathies some of which correlate with clinical severity. While there are consistent changes amongst these four disorders, there are also condition specific findings that need to be further refined and validated. Overall, these results provide a foundation for further refinement of these measures for use in future clinical trials for these conditions.

## Introduction

Developmental encephalopathy (DE) is an emerging term to group genetic neurodevelopmental disorders with overlapping clinical features [1]. These disorders are best exemplified by Rett syndrome (RTT), one of the first clinically identified neurodevelopmental disorders [2], caused by variants in the X-linked gene *MECP2* [3]. RTT has been historically linked to a number of other single-gene neurodevelopmental disorders due to shared clinical features, including CDKL5 deficiency disorder (CDD), MECP2 duplication syndrome (MDS), and FOXG1 syndrome (FOXG1). These shared features include epilepsy, autonomic system dysfunction, fine and gross motor impairment, sleep disturbances, social withdrawal, and intellectual disability. In addition to shared features, RTT, CDD, MDS, and FOXG1 have a few distinct developmental and clinical characteristics. For instance, compared to individuals with RTT, individuals with MDS tend to be male and are less likely to experience a period of early regression [4, 5]*.* Children with CDD are more likely than children with RTT, MDS, and FOXG1 to experience early-life epilepsy, with seizures occurring in most children by three months of age [6]. Cortical visual impairment is common in children with CDD and FOXG1 [6–9]. Clearly, both shared and discreet features exist in these related conditions.

There are currently no effective treatments for any of the DEs. However, continued progress is being made in the preclinical development of targeted therapeutics for the DEs, including gene-based therapies [10–13]. As these novel therapies move toward clinical trials, there is a pressing need for sensitive outcome measures of brain function to objectively and precisely evaluate clinical therapeutic efficacy.

Individuals with RTT are known to present with abnormalities on neurophysiological measures such as resting EEG and evoked potentials (EPs; [14, 15]). To identify whether such measures may be useful as outcome measures for RTT and related DEs, a multi-site study of EPs and resting EEG was conducted from 2017 to 2021 as part of the Natural History Study of Rett Syndrome & Rett-Related Disorders (NHS). Recent reports of the visual and auditory EPs from the RTT and CDD cohorts from the NHS have indicated that EP amplitudes correlate with clinical severity in these populations [16, 17]. Namely, in the RTT cohort from the NHS both VEP and AEP amplitudes negatively correlated with clinical severity [16]. In CDD, AEP amplitude was similarly more attenuated in individual’s with greater clinical severity [17]. This pattern of reduced EP amplitude with greater severity has also been reported by independent studies examining EPs in RTT [18, 19]. Together these findings provide initial evidence that EPs may be useful as objective measures of brain function in RTT and CDD and underscore the need for further research in this area.

The aim of the current study is to compare and contrast the EPs across all four DEs in the NHS including the MDS and FOXG1 groups. To our knowledge, no prior study has characterized EPs in MDS. However, studies that have considered other electrophysiological measures in MDS have suggested that RTT and MDS may be associated with distinct electrophysiological responses [20, 21]. Only one prior study has considered EPs in FOXG1. This study found no difference in the flash VEP of individuals with FOXG1 and TD individuals [8], although the generalization of these results were limited due to a small sample size. In addition to conducting group-level comparisons of EPs in RTT, CDD, MDS, and FOXG1, the current study also compares associations between the EPs and clinical severity within each of the four DEs. The existing analyses of the RTT and CDD cohorts have indicated that aspects of the EPs, particularly EP amplitudes, correlate with measures of clinical severity in RTT and CDD, with lower amplitudes in individuals with greater clinical severity. Therefore we set out to determine if similar or different aspects of the EPs would correlate with clinical severity in MDS and FOXG1.

## Methods

### Participants

All data were acquired as part of the NHS protocols 5211 and 5212 (U54 HD061222; ClinicalTrials.gov: NCT00299312/NCT02738281). The EPs were acquired at one of five sites: Boston Children’s Hospital (BCH), University of Colorado/Children’s Hospital Colorado (UC-CHCO), Children’s Hospital of Philadelphia (CHOP), Cincinnati Children’s Hospital (CCH), or Vanderbilt University Medical Center (VUMC). Data acquisition for each of the four DEs as well as the control group was distributed across the five sites. Eligibility for the RTT group included a documented pathogenic variant in *MECP2* and confirmed diagnosis of typical or atypical RTT based on consensus criteria [22]. Eligibility for the other clinical groups included a documented pathogenic or likely pathogenic variant in *CDKL5* (CDD group), a duplication encompassing *MECP2* (MDS group), or a deletion, duplication, or missense mutation encompassing or in *FOXG1* (FOXG1 group). All potential participants must have been also been enrolled in the clinical protocol of the NHS (5211). The experimental protocol was approved by the appropriate Institutional Review Boards of CHOP, VUMC, BCH, CCH, and UC-CHCO. For the NHS protocol (5211) the appropriate Institutional Review Boards of CHOP and VUMC approved the protocol, whereas UC-CHCO, BCH, and CCH relied on the single-IRB agreement provided by the University of Alabama at Birmingham. Written informed consent was obtained for each participant according to the Declaration of Helsinki. Data acquisition occurred between 2017 and 2021.

Overall, 80 individuals with RTT, 28 individuals with CDD, 17 individuals with MDS, and 12 individuals with FOXG1 participated in the study. Participants with RTT were older than the MDS, CDD, and FOGX1 participants, on average. To facilitate group comparisons, the current analyses were restricted to participants under 18 years of age (for analyses of the full RTT and CDD groups, see [16, 17]). A number of additional participants had to be excluded from each group due to poor data quality (defined as < 25% of trials accepted), absence of the expected predominant peak (P1 for VEP; N1 for AEP), or other factors (for details of exclusions, see Tables 1 and 2). Following exclusions, VEP data was available for 43 individuals with RTT (mean age = 7.8 years, SD = 4.5, range = 2.5–17.7), 16 individuals with CDD (mean age = 5.4 years, SD = 3.8, range = 1.7–15.7), 15 individuals with MDS (mean age = 7.7 years, SD = 4.5, range = 2.1–16.0), and 5 individuals with FOXG1 (mean age = 5.5 years, SD = 3.7, range = 1.4–10.1; Table 1). AEP data was available for 51 individuals with RTT (mean age = 8.9 years, SD = 4.7, range = 2.5–17.7), 14 individuals with CDD (mean age = 6.2 years, SD = 4.3, range = 1.7–15.7), 14 individuals with MDS (mean age = 7.6 years, SD = 4.6, range = 2.1–16.0) and 6 individuals with FOXG1 (mean age = 9.0 years, SD = 4.8, range = 3.4–17.0; Table 2).

**Table 1:** Demographics and exclusions: visual evoked potentials

	**TD**	**RTT**	**CDD**	**MDS**	**FOXG1**
**Participants meeting age criteria** (*n*)	26	66	25	16	11
**Total participants excluded** (*n*)	1 (4%)	23 (35%)	9 (36%)	1 (6%)	6 (55%)
Data quality	-	4	2	-	2
Absence P1	-	13	6	1	2
Other^a^	1	6	1	-	2
**Final Group** (*n)*	**25**	**43**	**16**	**15**	**5**
Age (years)	6.1 (5.9)	6.6 (6.3)	4.6 (4.1)	6.6 (8.1)	5.2 (7.2)
Males	9	1	5	13	3
Females	16	42	11	2	2
CSS	-	20 (11)	28 (10)	15 (20)	29 (21)
MBA	-	42 (16)	51 (20)	32 (31)	53 (45)
Seizure frequency	-	0 (1)	4 (2)	0 (1)	1 (4)

^a^Other reasons for exclusion were falling asleep, technical error, no VEP acquired due to early termination, and excessively slow background EEG; Values presented as median (IQR); Seizure frequency: 0 = absent, 1 = none with medications, 2 = monthly, 3 = weekly, 4 = daily. Age, CSS, MBA, and seizure frequency are presented as median (interquartile range)

**Table 2:** Demographics and exclusions: auditory evoked potentials

	**TD**	**RTT**	**CDD**	**MDS**	**FOXG1**
**Participants meeting age criteria** (*n*)	26	66	25	16	11
**Total participants excluded** (*n)*	3 (11%)	15 (23%)	11 (44%)	2 (12%)	5 (45%)
Data quality	1	7	2	0	5
Absence N1	1	4	9	1	0
Other^a^	1	4	0	1	0
**Final Group** (*n)*	**23**	**51**	**14**	**14**	**6**
Age (years)	6.1 (5.9)	8.9 (8.3)	5.6 (7.9)	6.5 (8.6)	9.2 (7.1)
Males	8	1	3	11	2
Females	15	50	11	3	4
CSS	-	19 (11)	26 (10)	15 (20)	26.5 (25)
MBA	-	44 (18)	48 (12)	31.5 (32)	49.5 (49)
Seizure frequency	-	0 (1)	3 (3)	0 (1)	0.5 (2)

^a^Other reasons for exclusion were falling asleep, technical error, no AEP acquired due to early termination, and excessively slow background EEG; Values presented as median (IQR); Seizure frequency: 0 = absent, 1 = none with medications, 2 = monthly, 3 = weekly, 4 = daily. Age, CSS, MBA, and seizure frequency are presented as median (interquartile range)

Twenty-five typically developing (TD) individuals with no known neurologic, neuropsychiatric, or genetic condition served as a comparison group (9 male; mean age = 7.5 years, SD = 4.0, range = 1.4–16.4; Table 1). Typical development was confirmed in all TD participants using methods described previously [16, 17]. One TD participant was excluded due to early termination/non-compliance (*n* = 1). Two additional TD participants were excluded from the analyses of the AEP only due to poor data quality (*n* = 1) or absence of an identifiable N1 component (*n* = 1). As designed, there was no significant difference in age between the RTT, CDD, MDS, FOXG1 and TD participants included in the analyses of the VEP (*H*(4) = 5.68, *p* = 0.225) or the AEP (*H*(4) = 4.95 *p* = 0.293). The TD participants data has been published previously in our full RTT and CDD cohorts [16, 17].

### Clinical measures

Two clinician-completed measures of clinical severity were available for all participants: Clinical Severity Score (CSS; [23, 24]) and Motor Behavioral Assessment (MBA; [23]). These assessments were created for RTT, but encompass the features common across the DEs including epilepsy and motor, cognitive, and autonomic disturbances. The CSS has 13 items with a maximum score of 58. The MBA has 34 items with a maximum score of 136. Higher scores represent greater disease severity. Seizure frequency was additionally considered for each participant and defined as an ordinal variable with five categories: absent, none with medications, monthly, weekly, or daily. This information was derived from a single item on the MBA that addressed seizure frequency.

### Stimuli

All sites followed standardized procedures for the VEP and AEP recording. The VEP stimuli consisted of 400 trials of a reversing black and white checkerboard presented continuously (0.5 cpd, 100% contrast, 2 Hz refresh rate). One study site (BCH) employed eye tracking (Tobii Technology, Danderyd, Sweden) to pause the visual paradigm when participants looked away from the stimulus. At the other sites, the stimuli ran continuously. An experimenter or parent was in the room with the child to redirect attention when necessary. Prior analyses have indicated similar findings between the site with eye-tracking and the sites using experimenter/parent redirection to the stimuli [16]. The AEP stimuli consisted of 520 trials of 500 Hz sinusoidal tones (300 ms duration) with a varying interstimulus interval of 0.6 to 2 s. The tones were presented at 60 dB SPL using a free-field speaker.

### EEG Methods

EEG equipment varied by site. At CCH, EEG was recorded from 21 individual Ag/AgCl electrodes (FPz reference) using a Natus EEG32U Amplifier (Natus Neuro, Middleton, WI, USA; 512 Hz SR). At CHOP, EEG was acquired using a 60-channel Ag/AgCl electrode cap (FCz reference) using the EEG amplifier of an Elekta VectorView (Elekta Oy, Helsinki, Finland; 1000 Hz SR). For the other sites (BCH, UC-CHCO, and VUMC), EEG was recorded from a 128 channel Electrical Geodesics Net (reference Cz) using a Net Amps amplifier (Electrical Geodesics, Inc, Eugene, OR, USA). Data was acquired in a shielded room at BCH and a non-shielded room at the other four sites. Electrode impedances were checked before all recordings and kept below the individual systems’ recommendations. To account for site differences in amplitude and latency of the EPs, data for all participants were adjusted prior to final analysis. Adjustments were based on data from a traveling, adult human phantom who completed the EP tasks at all study locations (for details of equipment and correction, see reference [16]).

Data analysis was performed at one central location (CHOP). Evoked potentials were analyzed in BESA (BESA 6.0 GMbH, Grafelfing, Germany) using methods described previously [16, 17]. Briefly, files collected at 1000 Hz were downsampled to 512 Hz. Bad channels and periods of excessive artifact were manually marked and excluded. Ocular artifacts were removed using automatic artifact correction methods in BESA. The artifact-corrected data were transformed to a reference-free, 81-channel array to allow for cross-site comparisons. The continuous files were then digitally filtered 3–40 Hz and segmented into 500 ms epochs for the VEP (-100 to 400 ms relative to stimulus onset) and 600 ms for the AEP (-150 to 450 ms). The segmented files were baseline corrected based on the mean of the pre-stimulus period. Epochs were excluded if the amplitude at any channel exceeded ± 250 μV. The number of accepted epochs for each group are provided in Table 1 (VEP) and Table 2 (AEP). A Kruskal–Wallis H test indicated a significant difference in the number of accepted epochs for VEP across groups (*H*(4) = 11.25, *p* = 0.024), however, all pairwise comparisons were insignificant with the adjusted α of *p* = 0.005. There was no difference in the number of accepted epochs across the five groups for the AEP (*H*(4) = 8.428, *p* = 0.077).

Analysis of the VEP focused on the N1, P1, and N2 components of the response at the midline occipital electrode (Oz). The P1 was defined as the first positive component closest to 100 ms. The N1 was defined as the negative peak immediately preceding the P1 and the N2 as the negative peak immediately following the P1. Analysis of the AEP focused on the P1, N1, and P2 components of the response at the frontal-central midline electrode (FCz). The N1 was defined as the first negative peak closest to 100 ms and the P1 and P2 were defined as the positive peaks immediately preceding and following the N1. Peak latencies and amplitudes were identified and measured using the peak finder in BESA. Interpeak amplitudes were measured peak to trough.

### Statistics

Statistics were performed in IBM SPSS Version 27. Non-parametric Kruskal–Wallis tests were used to assess group differences due to non-normality and unequal variances between groups. Kruskal–Wallis H tests were followed by paired comparisons with a Bonferroni-adjusted α of *p* = 0.005 (alpha of 0.05/10 comparisons = 0.005). Linear regression analysis was used to identify potential associations between age and VEP/AEP parameters for all groups and within each of the DEs, associations between the VEP/AEP parameters and clinical severity (CSS or MBA). Significance for linear regression analyses was set to *p* = 0.05. The log transformation (log10) of age was entered in all analyses using age to account for a positive skew in the age of the participants.

## Results

### Visual evoked potentials

#### Demographics and clinical variables

Demographics and clinical severity scores for participants included in the analysis of the VEP are provided in Table 1. There was a significant group difference in CSS (*H*(3) = 9.65, *p* = 0.022) with follow-up pairwise comparisons indicating greater severity for the CDD group compared to the MDS group *(p* = 0.005)*.* There were no significant group differences in MBA (*H*(3) = 7.38, *p* = 0.061). There was a significant group difference in seizure frequency (*H*(3) = 27.0, *p* < 0.001) with greater seizure frequency in participants with CDD compared to participants with RTT (*p* < 0.001) and MDS (*p* < 0.001).

### Group comparison

**Fig. 1:**
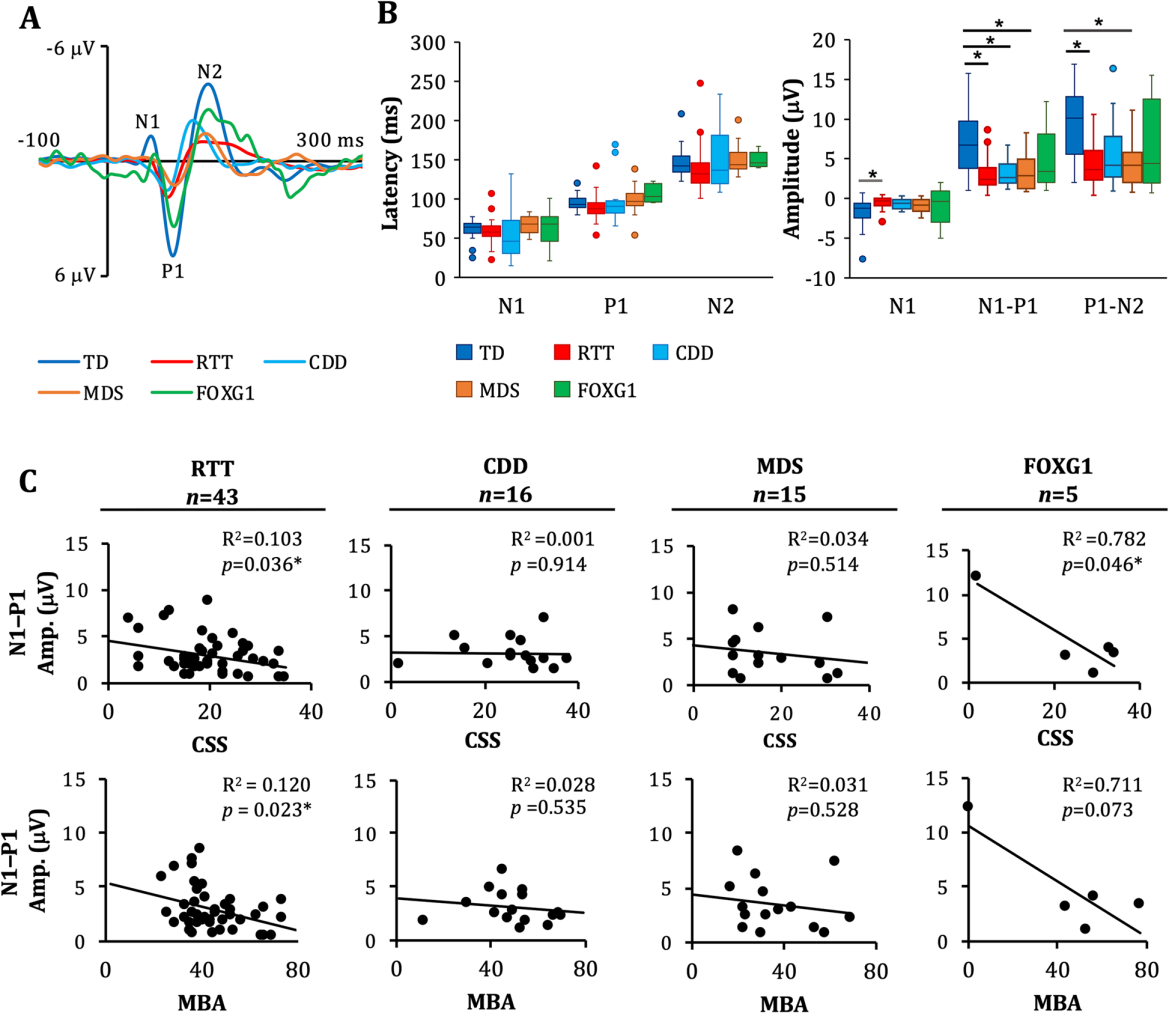
Visual Evoked Potentials. **A** Grand average VEP waveforms for TD, RTT, CDD, MDS, and FOXG1 groups at electrode Oz. **B** Box plots showing the distribution of the latency and amplitude of the VEP components for TD, RTT, CDD, MDS, and FOXG1 participants. The amplitude of multiple components was reduced in RTT, CDD, and MDS groups compared to the TD group. Statistical analyses were performed with Kruskal–Wallis tests and post-hoc tests analyses with Bonferroni-adjusted *p* values for significance (*p < 0.005). **C** Scatterplots showing the association between VEP N1-P1 amplitude and clinical severity (CSS and MBA) for RTT, CDD, MDS, and FOXG1 participants. N1-P1 amplitude was significantly associated with both severity measures in participants with RTT and FOXG1. No aspects of the VEP were significantly associated with severity for the other groups. Statistical analyses were performed using linear regression (*p < 0.05)

Kruskal–Wallis H tests indicated significant group differences in VEP N1, N1–P1, and P1–N2 amplitude (*H*(4) = 15.3, *p* = 0.004; *H*(4) = 24.4, *p* < 0.001; *H*(4) = 25.1 *p* < 0.001, respectively; Fig. 1A-B). Follow-up comparisons indicated reduced N1 amplitude in participants with RTT compared to TD participants (*p* < 0.001), reduced N1–P1 amplitude in participants with RTT (*p* < 0.001), CDD (*p* = 0.001), and MDS (*p* = 0.003) compared to TD participants, and reduced P1–N2 amplitude in participants with RTT (*p* < 0.001) and MDS *(p* < 0.001) compared to TD participants. There were no significant differences in VEP amplitude between the FOXG1 group and the other groups or significant differences in VEP latency for any of the groups (Table 3).

**Table 3:** Visual evoked potential latency and amplitude

	**TD** **(*n* = 25)**	**RTT** **(*n* = 43)**	**CDD** **(*n* = 16)**	**MDS** **(*n* = 15)**	**FOXG1** **(*n* = 5)**
N1 latency	64.0 (12.8)	58.4 (13.7)	46.5 (42.5)	68.1 (31.0)	68.0 (20.5)
P1 latency	93.4 (11.8)	87.5 (13.7)	90.6 (15.7)	97.4 (15.4)	103.1 (23.4)
N2 latency	142.3 (20.6)	132.4 (25.4)	137.3 (61.6)	144.1 (21.4)	146.2 (18.5)
N1 amplitude	-1.25 (1.8)	-.338 (.89)	-.588 (1.1)	-.809 (1.4)	-.412 (3.9)
N1-P1 amplitude	6.74 (5.9)	2.38 (2.2)	2.66 (2.4)	2.90 (3.7)	3.43 (6.1)
P1-N2 amplitude	10.1 (7.3)	3.60 (3.7)	4.19 (52)	4.15 (3.8)	4.4 (10.6)
Accepted trials	349 (77)	329 (106)	344 (65)	354 (51)	124 (169)

Data are presented as median (interquartile range). Latency values are in milliseconds. Amplitude values are in microvolts

### Associations with clinical severity: VEP amplitude

In participants with RTT, VEP N1 and N1-P2 amplitudes were significantly associated with clinical severity with decreasing amplitude with increasing severity (N1 amplitude & CSS: R^2^ = 0.096, *F* (1, 42) = 4.34, β = -0.309, *p* = 0.044; N1-P1 amplitude & CSS: R^2^ = 0.103, *F* (1, 42) = 4.72, β = -0.321, *p* = 0.036; N1-P1 amplitude & MBA: R^2^ = 0.120, *F* (1, 42) = 5.62, β = -0.347, *p* = 0.023). VEP N1-P2 amplitude was also negatively associated with CSS in participants with FOXG1 (R^2^ = 0.782, *F* (1, 4) = 10.77, β = -0.884, *p* = 0.046), however this association was largely driven by a single participant with milder symptoms (see Fig. 1C). Clinical severity using the current measures was not significantly associated with any of the VEP parameters for participants with CDD or MDS (Fig. 1C). Additional analyses were conducted to determine if the absence of a P1 was associated with greater clinical severity. This analysis demonstrated no difference in CSS or MBA score for participants who were excluded from the analysis of the VEP for the absence of a P1 component (*n* = 22), on average, compared to those participants with an identifiable P1 (*n* = 79; CSS: *p* = 0.133; MBA:* p* = 0.169).

### Associations with age

VEP N1-P1 and P1-N2 amplitudes decreased with age in participants with RTT (R^2^ = 0.115, β = -0.338, *p* = 0.026; R^2^ = 0.129, β = -0.359, *p* = 0.018, respectively). There were no significant associations between age and the VEP for the TD, CDD, MDS, or FOXG1 groups (Fig. 3).

### Auditory evoked potentials

#### Demographics and clinical variables

Demographics and severity scores for participants included in the analysis of the AEP are provided in Table 2. There were no significant group differences in overall severity on the CSS or MBA (*H*(3) = 4.42, *p* = 0.220; *H*(3) = 4.88, *p* = 0.181, respectively). There was a significant group difference in seizure frequency (*H*(3) = 20.9, *p* < 0.001) with pairwise comparisons indicating greater seizure frequency in CDD compared to RTT (*p* < 0.001) and MDS (*p* = 0.001).

### Group comparison

**Fig. 2:**
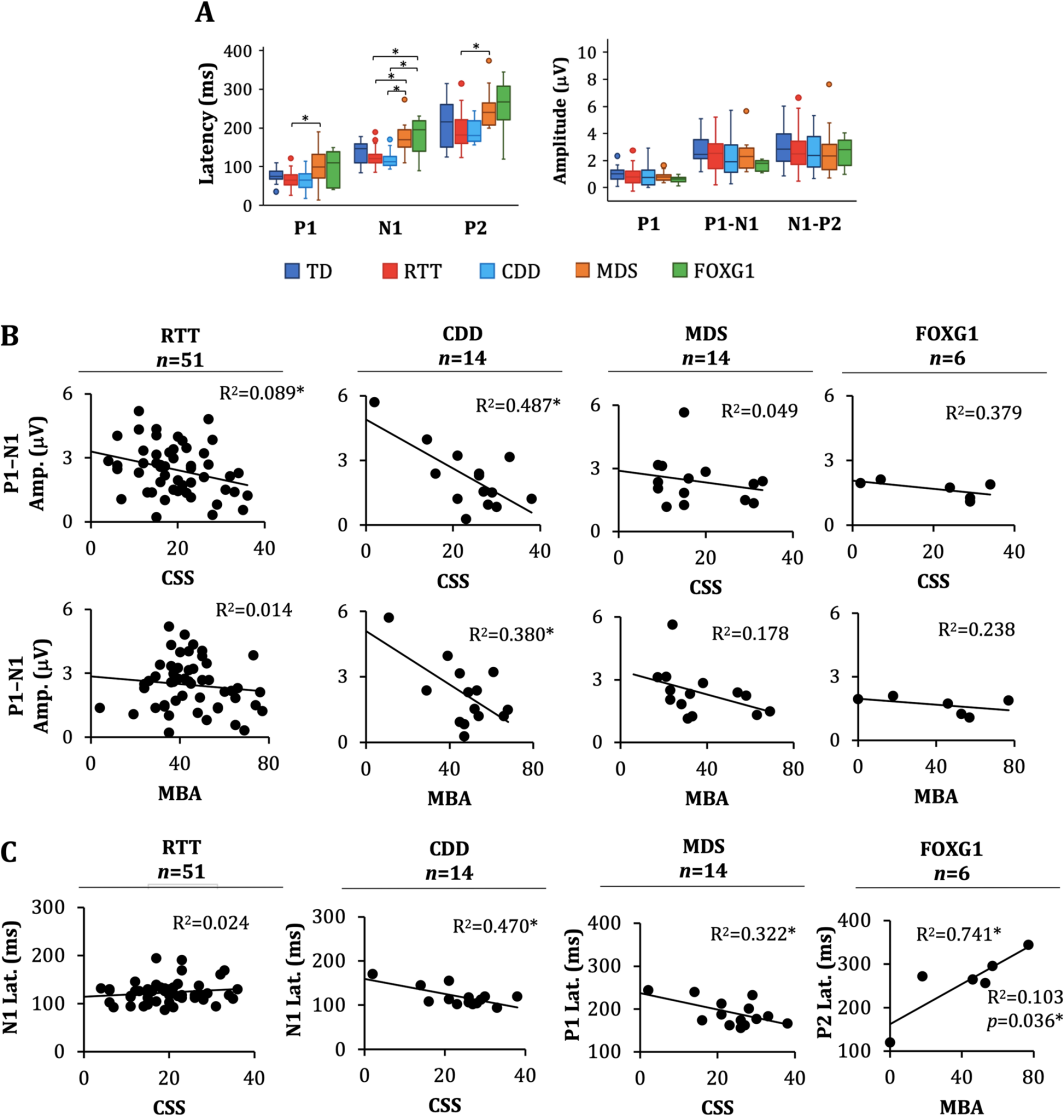
Auditory Evoked Potentials. **A** Box plots showing the distribution of the latency and amplitude of the AEP components for TD, RTT, CDD, MDS, and FOXG1 participants. The peak latencies of AEP components were prolonged in participants with MDS and FOXG1 compared to participants with RTT and CDD. Statistical analyses were performed with Kruskal–Wallis tests and post-hoc analyses with Bonferroni-adjusted *p* values for significance (*p < 0.005). **B** Scatterplots illustrating the association between AEP P1-N1 amplitude and the severity measures (CSS and MBA) for RTT, CDD, MDS, and FOXG1 participants. P1-N1 amplitude was significantly associated with severity in RTT and CDD. **C** Scatterplots showing the association between the latency of select AEP components and the clinical severity measures in participants with RTT, CDD, MDS, and FOXG1. The latency of one or more of the AEP components was significantly associated with severity in CDD, MDS, and FOXG1. Statistical analyses for **B-C** were performed using linear regression (*p < 0.05). The grand average AEP waveforms are not included due to age-related shifts in peak latency, particularly for the TD group, which obscure comparisons of peak amplitudes

There was a significant effect of group on AEP P1, N1, and P2 latency (*H*(4) = 15.4, *p* = 0.004; *H*(4) = 26.5, *p* < 0.001; *H*(4) = 11.1, *p* = 0.011, respectively; Fig. 2A). Follow-up pairwise comparisons indicated delayed P1 latency in MDS compared to RTT (*p* < 0.001), delayed N1 latency in MDS compared to RTT (*p* < 0.001) and CDD (*p* < 0.001), delayed N1 latency in FOXG1 compared to CDD (p = 0.003) and RTT (*p* = 0.005), and delayed P2 latency in MDS compared to RTT (*p* < 0.001). There were no group differences in the amplitude of the AEP components (Table 4).

**Table 4:** Auditory evoked potential latency and amplitude

	**TD** **(*n* = 23)**	**RTT** **(*n* = 51)**	**CDD** **(*n* = 14)**	**MDS** **(*n* = 14)**	**FOXG1** **(*n* = 6)**
P1 latency	74.6 (19.5)	64.8 (25.4)	65.8 (35.5)	99.9 (60.5)	109.8 (93.2)
N1 latency	146.8 (48.8)	121.4 (21.4)	112.7 (22.4)	169.3 (41.1)	195.7 (79.4)
P2 latency	215.2 (109.4)	182.0 (60.5)	180.8 (52.7)	240.5 (56.2)	267.9 (85.8)
P1 amplitude	1.04 (.65)	.793(.83)	.739 (1.1)	.806 (.40)	.650 (.30)
P1-N1 amplitude	2.44 (1.4)	2.52 (1.8)	1.92 (2.0)	2.29 (1.4)	1.80 (.77)
N1-P2 amplitude	2.85 (2.0)	2.48 (1.7)	2.37 (2.2)	2.32 (1.9)	2.80 (1.9)
Accepted trials	487.0 (60)	433.0 (130)	427.5 (182)	454.5 (95)	403.5 (277)

Data are presented as median (interquartile range). Latency values are in milliseconds. Amplitude values are in microvolts

### Associations with clinical severity: AEP amplitude

In participants with RTT, AEP P1 and P1-N1 amplitudes were associated with CSS with decreasing amplitude with increasing severity (P1 amplitude: R^2^ = 0.123, *F* (1, 44) = 6.89, β = -0.351, *p* = 0.012; P1 – N1 amplitude: R^2^ = 0.089, *F* (1, 50) = 4.78, β = -0.298, *p* = 0.034). AEP amplitudes were also negatively associated with severity in participants with CDD. Specifically, in participants with CDD, P1 and P1–N1 amplitudes were associated with both CSS and MBA (CSS: P1 amplitude: R^2^ = 0.455, *F* (1, 13) = 10.03, β = -0.675, *p* = 0.008; P1–N1 amplitude: R^2^ = 0.487, *F* (1, 13) = 11.41, β = -0.698, *p* = 0.005; MBA: P1 amplitude: R^2^ = 0.353, *F* (1, 13) = 6.53, β = -0.594, *p* = 0.025; P1–N1 amplitude: R^2^ = 0.380, *F* (1, 13) = 7.37, β = -0.617, *p* = 0.019). The association between N1-P2 amplitude and severity was specific to the CSS (R^2^ = 0.380, *F* (1, 13) = 7.36, β = -0.617, *p* = 0.019). There were no associations between AEP amplitude and clinical severity for participants with MDS or FOXG1 (Fig. 2B). Additional analyses were conducted to determine if the absence of an AEP N1 peak was associated with clinical severity. This analysis revealed that participants without a N1 component (*n* = 14) had more severe CSS, on average, compared to those with a N1 (*n* = 85; *p* < 0.001, η^2^ = 0.109). There was no group difference in MBA scores based on the presence or absence of a N1 (*p* = 0.116).

### Associations with clinical severity: AEP latency

In participants with CDD, N1 and P2 latencies were associated with CSS with decreasing latency with increasing severity (N1 latency: R^2^ = 0.470, *F* (1, 13) = 10.64, β = -0.698, *p* = 0.007; P2 latency: R^2^ = 0.322, *F* (1, 13) = 5.69, β = -0.567, *p* = 0.034; see Fig. 2C). P1 latency was also negatively associated with severity in participants with MDS (CSS: R^2^ = 0.360, *F* (1, 13) = 6.74, β = -0.600, *p* = 0.023; MBA: R^2^ = 0.388, *F* (1, 13) = 7.60, β = -0.623, *p* = 0.017). In participants with FOXG1, P2 latency was associated with MBA, with increasing latency with increasing severity (R^2^ = 0.741, *F* (1, 5) = 9.48, β = 0.861, *p* = 0.028; Fig. 2C). There was no association of latency with severity in the RTT cohort.

### Associations with age

**Fig. 3:**
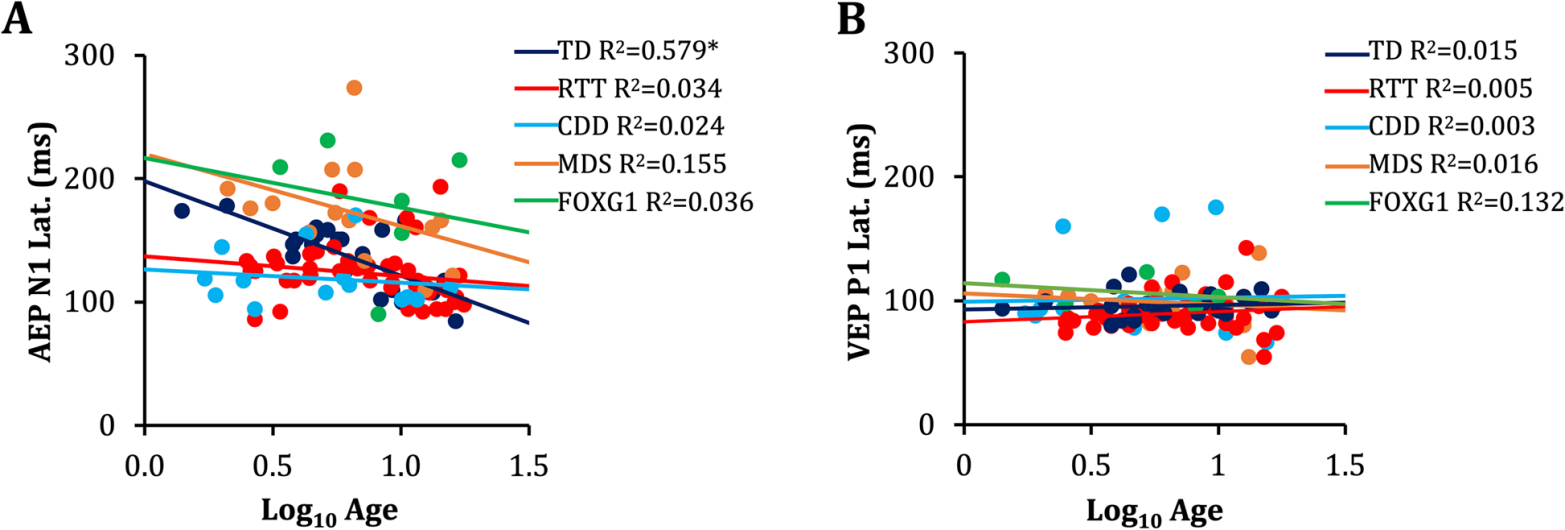
Associations between AEP and VEP Latency and Age for All Groups. **A** Scatterplot of AEP N1 latency and age in TD, RTT, CDD, MDS, and FOXG1 participants. N1 latency declined significantly with age in TD participants. Age was not significantly associated with AEP latency for participants with RTT, CDD, MDS, or FOXG1. **B** Scatterplot of VEP P1 latency and age for all groups. VEP latency did not change with age in any of the groups, consistent with the established literature on the stability of VEP P1 latency from early childhood. Statistical analyses for were performed using linear regression (*p < 0.05)

The latency of the AEP components declined with age in TD participants in line with the established literature on the typical maturation of the AEP (P1 latency: R^2^ = 0.420, *F* (1, 22) = 15.2, β = -0.648, *p* < 0.001; N1 latency: R^2^ = 0.579, *F* (1, 22) = 28.9, β = -0.761, *p* < 0.001; P2 latency,: R^2^ = 0.295, *F* (1, 22) = 13.7, β = -0.544 *p* = 0.007). AEP P1-N1 amplitude also decreased with age in TD participants (R^2^ = 0.179, *F* (1, 22) = 4.59, β = -0.423, *p* = 0.044). There were no associations between age and the AEP in participants with RTT, CDD, MDS, or FOXG1 (Fig. 3).

## Discussion

The current study directly compared visual and auditory EPs across four DEs with distinct and overlapping features. These data were acquired as part of the multi-site NHS of RTT, CDD, MDS, and FOXG1. Data from the RTT and CDD cohorts of the NHS have been provided previously [16, 17]. The current study extended these previous analyses to include the MDS and FOXG1 groups from the NHS and examined the association between the EPs and clinical variables across all four DEs.

Overall, the current results confirm and build on existing studies of EPs in RTT and CDD, including the prior reports of EPs in RTT and CDD from the NHS [16, 17]. Specifically, analyses of the VEP revealed a reduction of VEP amplitude in participants with RTT, CDD, and MDS compared to TD participants. This is consistent with other recent studies of the VEP in RTT and CDD [16–18] and underscores the potential for the VEP to serve as a biomarker of brain function across different DEs. Further pointing to the potential of the VEP as a biomarker, the extent of attenuation in VEP amplitude was significantly associated with clinical severity in participants with RTT, with a reduction in VEP amplitude with increasing clinical severity. This result is consistent with the analyses of the full RTT cohort from the NHS [16]. VEP amplitude was also negatively associated with severity in FOXG1. However, this effect was driven primarily by a single participant with lower-than-average severity, and therefore future work with a larger sample size is needed to confirm the presence of an association between clinical severity and VEP amplitude in FOXG1. No aspects of the VEP were significantly associated with severity in participants with CDD or MDS, which may be due in part to the relatively smaller sample sizes and/or use of clinical severity scales that were designed for RTT and may not fully capture symptom severity for these other conditions. In participants with CDD, the absence of an association between the VEP and clinical severity may also be related to the prevalence of cortical visual impairment in this population. Estimates of CVI were not available for the current participants, but future studies should include measures of CVI as a potential mediating variable between the VEP and clinical severity, particularly given the prevalence of CVI in CDD and FOXG1.

In contrast to the VEP, there were no significant group differences between DE and TD participants in the AEP. This was surprising given prior studies reporting reduced AEP amplitude in participants with RTT compared to TD participants [16, 19, 25, 26]. The lack of a group difference in AEP amplitude between RTT and TD participants in the current analysis may be due to a focus on younger children whereas prior studies have included both younger and older individuals. Indeed, in the analyses of the full cohort of participants with RTT from the NHS, AEP amplitude declined significantly with age [16], an effect that was not replicated with the smaller age-range used here. Despite no group-level differences in AEP amplitude, the amplitude of specific AEP components correlated with clinical severity within the RTT and CDD groups, which is consistent with the prior analyses of the full RTT and CDD cohorts from the NHS [16, 17].

Although there were no significant differences in the AEP between the TD and DE groups, there were significant differences in the AEP between the four DEs with prolonged AEP latencies in MDS and FOXG1 compared to RTT and CDD. The latency of specific components of the AEP also correlated with severity measures in CDD, MDS, and FOXG1. In FOXG1, longer latencies were observed in more severely affected individuals. In CDD and MDS, the opposite pattern was observed, with longer latencies in participants with milder symptoms. This was unexpected, given longer latencies have been associated with more severe cognitive impairment in other neurodevelopmental disabilities [27, 28]. Given the small sample sizes available here, future work is needed to confirm these finding and further elucidate the association between AEP latency and clinical severity in these populations. Nonetheless, the current results provide the first report that AEP latencies are prolonged in individuals with MDS and FOXG1 and thus, AEP latency may be meaningful as an electrophysiological measure of disease evolution and severity in these populations.

The existing analyses of the full RTT cohort from the NHS included participants up to 37 years of age [16]. The current analysis was restricted to younger participants (< 18 years) due to the absence of adult participants with MDS and FOXG1 in the dataset. The results from the analyses of full RTT cohort were largely consistent with the results presented here. Namely, VEP and AEP amplitudes were negatively associated with clinical severity in both the full cohort and the younger cohort here, suggesting that the same EP parameters may be useful as outcome measures across different developmental stages. One open question that was not addressed in the current analysis or in prior analyses of the EPs from the NHS [16, 17] concerns the timeline for when evoked potentials first become abnormal. This question could be addressed by future studies with children recently diagnosed or showing signs of these disorders. Given the early onset of CDD and FOXG1, this would require studies with infants. For RTT, such studies would be with slightly older children who are just beginning to show signs of regression.

### Limitations

There are a number of limitations of the current study that will need to be addressed by future work. A number of participants had to be excluded due to excessive artifact or absence of the expected predominant peaks. The percentage of excluded participants was particularly high for the FOXG1 group, with exclusion rates of ~ 50% for the VEP and AEP. The current study employed traditional approaches for pre- and post-processing of EPs to facilitate comparisons to the larger literature on EPs. However, the current rates of exclusion indicate that these methods are not optimal for analyzing EPs in these populations, particularly in participants whose recordings contain a high-degree of artifact and/or whose waveforms do not conform to the patterns expected based on the waveforms of TD individuals. Therefore, a main focus of future work should be to identify novel approaches for reducing artifact and quantifying EPs in individuals with DEs. A related issue concerns the reproducibility of EPs in these populations. Although not addressed in the current paper, the analyses of the full RTT and CDD cohorts indicated low intersession reproducibility for some participants [16, 17]. Future work aimed at identifying novel acquisition and analysis methods for EPs in the DEs must also consider ways to optimize reproducibility of the responses.

Due to the number of participants that had to be excluded, the sample sizes were relatively small (with exception of RTT group) and may have been insufficiently powered to detect group differences and associations with the clinical variables. This is particularly true for FOXG1 group, which was limited to 5 and 6 participants for the analysis of the VEP and AEP, respectively. Despite these small sample sizes, a number of findings for the FOXG1 group were significant. Further work with a larger sample size is needed to confirm these patterns and provide a greater understanding of EPs in this population. A larger group would also allow analyses of other variables that we were too low powered to study here, including the impact of seizure medications.

Existing studies of the VEP in RTT and CDD have demonstrated an attenuation of the VEP with or without eye tracking [17, 18]. In this study, only one site employed eye tracking, therefore, future studies are needed to elucidate the effect of attention on the VEP in this population in particular, and determine the best approach for minimizing attention differences as a potential source of noise in the analyses. Although eye tracking may provide a precise measure of attention, it requires specialized equipment that may not be available at all sites, particularly in a distributed clinical trial and the effectiveness of the tracking in this impaired population needs further evaluation. Alternative approaches, such as manual triggering of the stimuli may be more feasible for this work moving forward.

## Conclusion

Despite these limitations, the current study provides new insights into EPs across different DEs, including the first report of both visual and auditory EPs in MDS and FOXG1. Overall, there was a degree of similarity across the DEs. There were robust differences in VEP amplitude between the RTT, CDD, and MDS groups compared to TD participants. There were no clear differences in the AEP between the DE and TDs, but within the DEs, aspects of the AEPs correlated with severity in all four DEs, pointing to the potential of the AEP as a biomarker at the individual level. The current study further demonstrates the utility of a multi-site approach and provides a foundation for further characterization and refinement of these measures as potential biomarkers of disease evolution, severity, and treatment response for the DEs. A major goal of future work in this area should be to refine methods for reducing noise and increasing signal of the EPs in this population to facilitate the feasibility of these measures for clinical trials.

## Data Availability

The EEG raw data is available upon request. The clinical variables and the Evoked potential summary variables are in process of being available on NIH dbGAP or are available upon request.
